# Supplementary material for: Go Wish Card Game for Meaningful Conversations in the Oncology Healthcare Context: A Narrative Review
Source: Cancers (Basel). 2025 Feb 7;17(4):560. doi: 10.3390/cancers17040560 (PMC11853492; doi:10.3390/cancers17040560)
Supplement: Supplementary file 1 [file cancers-17-00560-s001.zip › cancers-3278738-supplementary/cancers-3278738-supplementary.pdf]

To identify the main scientific evidence supporting the use of the GWCG, we conducted a search in August 2024 for published works in the following databases: PubMed, Lilacs, CINAHL, PsycINFO, and Embase. Keywords used included (but were not limited to): games, Go Wish, patient card games, advance care planning, palliative care, and supportive care. Additionally, a snowballing method of manual tracking through reference lists of previously selected articles was employed as a secondary search strategy. Based on titles and abstracts, relevant publications were retrieved in full for evaluation through a double-check reading methodology by two independent reviewers.

Thus, 40 articles related to the GWCG were selected, with the majority being qualitative studies (14 out of 40), followed by review articles (8 out of 40), observational studies (4 out of 40), and methodological studies (5 out of 40), which generally have lower generalizability and evidence levels compared to intervention studies. Intervention studies, in turn, constituted only 7.5% of the total articles (3 out of 40), reflecting the need for more clinical trials on the subject (Supplementary Table 1).

Supplementary Table S1: Main scientific evidence supporting the use of the Go Wish Cards Game.

| **Author (Year), Journal, Country** | **Type of Study** | **Objective** | **Patient Profile** | **Practical results** |
|---|---|---|---|---|
| **Qualitative Studies (n=14)** | | | | |
| Paiva BSR, et al. (2024) [11]<br>Ann Palliat Med<br>Brazil | Exploratory and descriptive study, part of a before-and-after clinical trial | To investigate the most significant EoL wishes of patients with cancer in palliative care.<br>. | 15 patients with cancer in PC. | - GWCG is a user-friendly, understandable, and flexible tool that can contribute to discussions about values and preferences in EoL.<br>- With a person-centered care framework and the support of a multidisciplinary team and family members, its use can help fulfill patients' wishes |

| | | | | |
|---|---|---|---|---|
| Johansson T, et al. (2022) [12]<br>BMC Geriatr<br>Switzerland | Study with participatory action research. | To explore the use, usability, and impact of the GWCG in discussions about EoL care at home. | 13 adults and elderly participants. | - GWCG can be used in a home setting.<br>- GWCG facilitated conversations about EOL values, influencing both the content of the discussion and the interactions, thereby supporting the preparation of ACP. |
| Ohnuki Y, et al. (2022) [13]<br>Tokai J Exp Clin Med<br>Japan | Study with participatory action research. | To determine whether the participants of a 'café-style' event (about ACP) implemented the ACP after the event | 14 adults and elderly participants. | - Participation in 'café-style' events may facilitate the implementation of ACP.<br>- Individuals may feel more comfortable discussing ACP with their family members than with their doctors. |
| Tishelman C, et al. (2022) [14]<br>Death Stud<br>Sweden | Study with participatory action research. | To develop and use a structured ACP approach based on conversations among elderly residents of Sweden. | 65 elderly participants. | - GWCG catalyzed discussions about EoL preferences.<br>- GWCG cards were considered a viable tool to stimulate person-centered conversations about preferences for future EoL care. |
| Eneslätt M, Helgesson G, Tishelman C. (2021) [15]<br>Palliat Care Soc Pract<br>Sweden | Study with Ripple Effect Mapping | To explore how GWCG were disseminated and used publicly to understand their impact on the community | 20 adults and elderly participants. | - GWCG were adapted for individual and group use, considering different settings.<br>- They were useful as an icebreaker to start conversations, to stimulate reflections on EoL with individuals with serious illnesses, and to strengthen professional relationships. |

| | | | | |
|---|---|---|---|---|
| Kroik L, et al. (2021) [16]<br>Scand J Caring Sci<br>Sweden | Exploratory and Descriptive Study | To investigate whether the Swedish adaptation of the GWCG supports discussions of EoL care preferences among the Sámi indigenous people. | 31 adults and elderly participants from the Sámi indigenous community. | - GWCGs are easy to use, comprehensible, flexible for initiating conversations, and beneficial in supporting discussions about values and EoL preferences. |
| Eneslätt M, Helgesson G, Tishelman C (2021) [17]<br>BMC Palliative Care<br>Sweden | Mixed Methods Study | Exploring the priorities and reasoning of elderly individuals regarding EoL care over time. | 52 elderly participants. | -The simultaneous approach based on conversations and the use of GWCGs for ACP is more suitable than using the cards alone for discussions about EoL preferences over time. |
| Eneslätt M, Helgesson G, Tishelman C (2020) [18]<br>Gerontologist<br>Sweden | Participatory Action Research Study | To explore the reasoning of elderly individuals in communities regarding their EoL care preferences in ACP. | 26 adults and elderly participants. | - While individuals may share some common preferences regarding EoL care, there can be variation in how they explain these preferences.<br>- These in-depth descriptions of reasoning are important for understanding the nature of EoL preferences. |

| | | | | |
|---|---|---|---|---|
| Möller UO, et al. (2020) [19]<br>J Hosp Palliat Nurs<br>Sweden | Content Analysis Study | To explore the experiences of patients in palliative care using GWCGs to discuss their wishes and priorities. | 40 patients in inpatient or home-based palliative care. | - Evaluation of the content of the cards (no participant found the content offensive or repulsive), sorting the cards (the sorting before the interview was a positive experience).<br>-Awareness of wishes that had not yet been discussed, sharing desires and priorities, reflecting on whether wishes and priorities change as death approaches.<br>- Patients were also able to identify not only what was important but also what was most important to prioritize in EoL care. |
| Thompson S, et al. (2019) [20]<br>Rural Remote Health<br>Australia | Qualitative Study | Understanding the perspectives and experiences of Aboriginal peoples in Western Australia for discussions on EoL. | 10 aboriginal participants (eight with cancer and two women from the Aboriginal cancer and EOL support group). | Community consultations raised discussions about wills, place of death, burial versus cremation, and the cost of funerals.<br>The use of cards facilitated discussions about personal wishes in EoL, including preferences regarding funeral details.<br>There are gaps in how conversations about EoL can be implemented in more diverse socio-cultural contexts. |

| | | | | |
|---|---|---|---|---|
| Lee MC, Hinderer KA, Alexander CS. (2018) [21]<br>Gerontology and Geriatric Medicine<br>USA | Focus Group Study | Description of the Most Important EoL Wishes (using GWCG) and the Concept of a Good Death for Sino-Americans | 60 Sino-American Patients | Main EoL Wishes:<br>- Physical Needs: Not feeling pain, trusting my doctor.<br>- Psychosocial Needs: Not being a burden to my family, having my family with me, maintaining my dignity.<br>-Spiritual Needs: Praying. |
| Osman H, et al. (2018) [22]<br>BMJ Support Palliat Care<br>Lebanon | Study with Thematic Analysis | Reporting the Experience with the Use of GWCG in Medical Education | 99 Medical Students | - The use of GWCG demonstrated that individuals have different priorities EoL care.<br>- Highlighted the importance of respecting patients' priorities and providing personalized care.<br>- Encouraged self-discovery and reevaluation of previous experiences.<br>- Transformed perspectives on death and dying.<br>- Increased appreciation for the importance of palliative care. |

| | | | | |
|---|---|---|---|---|
| Litzelman DK, et al. (2017) [23]<br>J Community Health<br>USA | Mixed Methods Study | Reporting How Trained CHWs Used GWCG to Identify Patient Preferences and Evaluate Whether Engaging in Conversations About ACP Was Associated with Subsequent Healthcare Utilization. | - Patients: elderly participants with chronic and multiple conditions, with dementia and/or depression.<br>- 392 participants (quantitative phase)<br>- 15 participants (qualitative phase) | - The use of GWCG by CHWs was helpful and positive in stimulating discussion on previously unaddressed issues.<br>- The three main card options chosen by patients were about spiritual and religious concerns, EoL preparation, and maintaining personal integrity. |
| Litzelman et al. (2016) [24]<br>Palliat Support Care<br>USA | Study with participatory action research | To provide education and training for patients, healthcare professionals, and organizations to facilitate EoL conversations. | 5,000 participants (community members, CHWs, healthcare professionals). | - Participants' perceptions of the quality and usefulness of the educational events varied from effective to very effective.<br>- Project based on: community health education, interprofessional team education, communication skills development, personal and professional renewal, and organizational change. |
| **Observational studies (n=4)** | | | | |
| Santana SCG, Câmara SB (2022) [25]<br>Rev Bras Cancerol<br>Brazil | Cross-sectional study. | To evaluate the perceptions and expectations of cancer patients regarding advance directives | 346 cancer patients | The GWCG facilitated conversations about advance directives and prompted reflection on EoL care. |
| Li T, et al. (2021) [26]<br>Am J Hosp Palliat Med<br>China | Cross-sectional study. | To evaluate EOL preferences among cancer patients using the HHG. | 58 cancer patients. | - The HTH can be used as a communication tool to encourage discussions about EOL care between cancer patients and healthcare professionals. |

| | | | | |
|---|---|---|---|---|
| Villavicencio-Chávez C, et al. (2019) [27]<br>Med Paliat<br>Spain | Cross-sectional study. | To explore the wishes of patients with ACD and CCD using the GWCG, from the perspective of family members. | 19 family members of patients with ACD and CCD. | - The GWCG stimulates conversations about EOL care between family members and the healthcare team, contributing to improved coping and reduced emotional burden for both. |
| Lankarani-Fard A, et al. (2010) [28]<br>J Pain and Symptom Manage<br>USA | Cross-sectional study. | To evaluate the feasibility of using the GWCG with seriously ill patients in the hospital. | 33 patients admitted to a veterans' hospital. | Most frequently mentioned wishes:<br><br>-Not feeling pain: should be addressed by the physician;<br><br>-Being at peace with God and praying: importance of spirituality and faith.<br><br>-The use of the GWCG in an inpatient setting is feasible. |
| **Intervention studies (n=3)** | | | | |
| Du J, et al. (2022) [29]<br>Int J Environ Res Public Healt<br>China | Randomized controlled clinical trial | To determine the effectiveness of using HTH in patients with advanced cancer in home PC. | 66 patients with advanced cancer in PC. | - The use of HTH reduces barriers to conversations about EoL care and may help patients maintain a more stable mental state. |
| Delgado-Guay MO, et al. (2016) [30]<br>Support Care Cancer<br>USA | Intervention study - Randomized clinical trial | To determine the EoL wishes of patients with advanced cancer and compare patient preferences between GWCG and LOS. | 100 patients with advanced cancer. | - EoL discussions using GWCG did not increase anxiety and were beneficial.<br>-The most important wishes expressed were: being at peace with God, praying, having my family with me, and not feeling pain. |

| | | | | |
|---|---|---|---|---|
| Wagner CD, et al. (2016) [31]<br>Am J Hosp Palliat Med<br>USA | Intervention study (Single-arm pilot study) | To test the feasibility and preliminary efficacy of a couple-based intervention with personalized activities (pre- and post-intervention outcomes for anxiety and depression). | 12 couples, with patients diagnosed with lung or breast cancer. | - 4 psychotherapy sessions, the second session used GWCG.<br>- Of the 2 patients identified with depression pre-intervention, 1 maintained the condition post-intervention. No changes were observed in the cases of anxiety (3 cases).<br>- Partners: lower HADS scores post-intervention.<br>- The interventions addressed existential concerns directly related to the safety and support of a close, loving relationship. |
| **Methodological studies (n=5)** | | | | |
| Dupont C, et al. (2022) [32]<br>BMC Public Health<br>Belgium | Development study (cultural adaptation) and pre-test. | To make cultural adaptations to the GWCG. | 12 healthcare professionals (consensus) and 33 adults and elderly individuals (pre-test). | - Sixteen of the 36 cards were adjusted. Three new cards were added: two with statements aligned with the rights of Belgian patients and euthanasia legislation, and one additional Wild Card.<br>-The cards supported conversations about EOL. |
| Lefuel P, et al. (2022) [33]<br>Nephrol Ther<br>Switzerland | Study of development and pre-testing. | To develop an ACP intervention for dialysis patients using the GWCG. | 12 dialysis patients. | - Interventions based on the GWCG can be created.<br>- The reflection process triggered by such interventions can lead the individual to introspection aligned with their deepest thoughts and allow for the choice of treatments that best meet their needs. |
| Perin M, et al. (2022) [34]<br>J Palliat Med<br>Italy | Development study (translation and cultural adaptation) and pre-testing | To develop an Italian version of the GWCG with linguistic translation and cultural adaptation. | 13 healthcare professionals and representatives from local associations of patients with serious illnesses. | The final version of the GWCG obtained contains various cultural peculiarities, with fewer explicit statements about EOL choices and a broader emphasis on the role of healthcare professionals in this discussion. |

| | | | | |
|---|---|---|---|---|
| Jia Z, et al. (2021) [35]<br>Am J Hosp Palliat Care<br>USA | Development study (cultural adaptation) and pre-testing. | To develop a culturally adapted ACP tool for Chinese Americans. | 2,267 Chinese American adults. | - The formats included individual and group styles.<br>-Participants found the session enjoyable (99.5%) and, after it, expressed an intention to comply with the ADs (86.5%). |
| Liu M, Chi I (2021) [36]<br>J Ethn Cult Diversit Social Work<br>China | Development study and pre-testing. | To develop a death education program ["My Life, My Wishes" (with HTH and other tools)] and test its practical implications. | 12 healthcare professionals/researchers (development) and 4 seniors (pre-testing). | Through death education programs, it is expected that seniors will improve communication with their families and healthcare professionals, rediscover their life experiences to live in a more meaningful way, and become familiar with EoL care. |
| **Review studies (n=8)** | | | | |
| Riley SR, et al. (2024) [37]<br>Palliat Care Soc Pract | Scope review | To synthesize ACP tools, highlighting their characteristics and methods. | 160 articles. | The GWCG was considered one of the most interactive methods for ACP. |
| Fernandes CS, Vale MB, Lourenço M. (2023) [38]<br>Palliat Support Care | Scope review | To identify and map the evidence from PC games | 53 articles. | - Card games are important for discussing EOL care, as they provide space for the expression of emotions and creativity.<br>- They can be applied to patients, family members, and caregivers, allowing them to talk about serious topics while playing. |
| Lourenço CG, Fernandes CS, Vale MBRC. (2023) [39]<br>Int J Palliat Nurs | Scope review | To examine the utility of games for nurses in PC | 17 articles. | -The advantages of using games include: improved knowledge about PC, enhanced communication skills, reduction of negative emotions, and increased abilities of the multidisciplinary team.<br>-They are effective and innovative pedagogical techniques in PC. |
| Liu L, et al. (2021) [40]<br>Palliat Med | Systematic review and meta-analysis. | To integrate quantitative and qualitative evidence to understand the effectiveness and experience of games for ACP. | 11 articles. | -The GWCG is an easy game with clear instructions that facilitates patient discussions about EoL issues and the expression of their preferences. |

| | | | | |
|---|---|---|---|---|
| | | | | - It is recommended by patients themselves, who found that the thought-provoking questions in the game provided a good opportunity to practice delicate conversations in ACP. |
| Meier EA, et al. (2016) [41]<br>Today's Geriatric Medicine | Integrative review | To suggest central themes related to the elements of a "good death." | N/A | -GWCG is one of the tools to guide personalized ACP.<br>-The use of GWCG has shown improved communication and discussion of important conversations among family members, as well as with the healthcare team.<br>- GWCG has demonstrated utility in resolving unfinished business and assisting in substitute decision-making about EoL care. |
| Barnes S, et al. (2012) [42]<br>J Pain and Symptom Manage | Integrative review | To identify communication interventions between patients and professionals developed for life-limiting conditions.<br><br>To explore their applicability in cancer patients and other groups. | 16 articles | - Central themes: use of education to improve professional communication skills, use of communication to enhance patient understanding, and use of communication skills to facilitate ACP.<br>- Interventions should include combined components of training, discussion with patients, education, and written communication. |
| Waldrop DP, Meeker MA. (2012) [43]<br>Nurs Outlook | Integrative review | To conduct a literature review on advance directives, ACP, and communication in EoL. | N/A | One of the representative intervention tools to improve communication and ACP in EoL is the GWCG. |
| Menkin ES. (2007) [44]<br>J Palliat Med | Integrative review with case reports | To describe the development of the GWCG and report on some of the various cases in which they were useful. | Report on 7 cases | - GWCG cards have been beneficial in promoting conversations between patients, their loved ones, and healthcare professionals.<br>- It is a useful, inexpensive, and intuitive tool for facilitating discussions about EoL. |

| | | | | |
|---|---|---|---|---|
| **Opinion article (n=2)** | | | | |
| Azizuddin DR, Thomas TH. (2022) [45]<br>JCO Clin Cancer Inform | N/A | To comment on the advancement of interventions with games in PC. | N/A | Games present complex concepts through engaging and illustrative representations, offering distraction, knowledge, entertainment, and social connection. They are promising, evidence-based, and accessible tools. |
| Menkin ES. (2010) [46]<br>J Palliat Med | N/A | To report the experience of preferences regarding EoL through the GWCG. | Hospitalized elderly patient due to probable ischemic colitis. | Addressing EoL wishes through the GWCG helps family members support patients' decisions. |
| **Theses and Dissertations (n=3)** | | | | |
| Caldwell A (2022) [47]<br>Georgia College<br>Georgia | Literature review and pilot project of a before-and-after clinical trial | To evaluate whether the GWCG facilitates conversations about EOL care.<br>To implement the GWCG in home-based palliative care and determine if it will facilitate EoL discussions. | 15 articles (review).<br>14 patients with neurological disease and cancer (clinical trial) | - GWCG can be used in different settings.<br>- This tool promotes the expansion of conversations about EOL care, helping patients determine what is important to them.<br>- It supports an increase in patient autonomy and the documentation of desires and values in EoL care records. |
| Gómez KMO. (2019) [48]<br>Universidad de San Carlos de Guatemala.<br>Guatemala | Cross-sectional study | To identify the needs and wishes of patients in palliative care through the GWCG. | 70 pacients in PC. | The use of the GWCG was important for identifying the needs and wishes of patients in palliative care, particularly the desire to avoid pain and to be at peace with God. |
| **Case Study (n=1)** | | | | |
| Jersak T, Gustin J, Humphrey L (2018) [49]<br>Journal of Palliative Medicine<br>USA | Case study | To report the experience of decision-making discussions using the GWCG in a patient with CHD. | 50-year-old man with CHD since childhood. | - The GWCG facilitated the initiation of conversations about what was important to the patient and his treatment goals.<br>- It promoted comfort in expressing his feelings, needs, and concerns, as well as trust in the healthcare team. |

| Londitudinal Study (n=1) | | | | |
|---|---|---|---|---|
| Potthoff M, (2017) [50]<br>South Dakota State University<br>USA | Mixed methods, longitudinal study | To describe the experience of parents of children in pediatric palliative care and the relationships between this experience and the characteristics of the illness.<br><br>To identify the effect of GWCG Pediatrics on parents and describe their experience using it. | 10 participants (parents of children in palliative care) | - The use of GWCG Pediatrics stimulated the translation of thoughts into action, empowered participation in conversations, and organized thoughts in the face of technical terms and concerns (described as "a beacon in the fog").<br>- GWCG Pediatrics can also support communication between spouses and the healthcare team. |

Note: PC= Palliative Care; EoL= end of life; GWCG= Go Wish Card Game; ACP= Advance Care Planning; ACD= Advanced Chronic Disease; CCD= Complex Chronic Disease; ADW= Anticipated Directives of Will; LOS= List of Wishes/Statements; NA= Not Applicable; HTH= Heart to Heart; CHD= Chronic Heart Disease; CHW= community health workers; HR= hazard ratio; CI=confidence interval; HCR= healthcare representatives; LOS= List of Wishes/ Statements; HADS= Hospital Anxiety and Depression Scale.
